# Alcohol’s Effects on Female Reproductive Function

**Published:** 2002

**Authors:** Mary Ann Emanuele, Frederick Wezeman, Nicholas V. Emanuele

**Affiliations:** Mary Ann Emanuele, M.D., is a professor in the Department of Medicine and in the Department of Cell Biology, Neurobiology, and Anatomy at Loyola University Stritch School of Medicine, Maywood, Illinois. Frederick Wezeman, Ph.D., is a professor in the Department of Orthopedic Surgery and Rehabilitation, and in the Department of Cell Biology, Neurobiology, and Anatomy; he is also Director of the Musculoskeletal Biology Research Lab at Loyola. Nicholas V. Emanuele, M.D., is a professor in the Department of Medicine at Loyola and a staff physician at the Veterans Affairs Hospital, Hines, Illinois

**Keywords:** reproductive effects of AODU (alcohol and other drug use), reproductive function, female, hypothalamic-pituitary-gonadal axis, hormones, puberty, postmenopause, menstrual cycle, osteoporosis

## Abstract

Mild-to-moderate alcohol use has numerous negative consequences for female reproductive function. Animal studies have shown that alcohol consumption disrupts female puberty, and drinking during this period also may affect growth and bone health. Beyond puberty, alcohol has been found to disrupt normal menstrual cycling in female humans and animals and to affect hormonal levels in postmenopausal women. Research has explored the mechanisms of these effects and the implications of these effects for bone health.

Mild-to-moderate alcohol use affects female reproductive function at several stages of life. It has been shown to have a detrimental effect on puberty, to disrupt normal menstrual cycling and reproductive function, and to alter hormonal levels in postmenopausal women. In addition, alcohol use can have implications for bone health. Before examining alcohol’s effect on female reproduction and the potential mechanisms of these effects, this article reviews normal female reproduction, including puberty, the normal female cycle, and hormonal changes in post-menopausal females.

## Overview of the Female Reproductive System

**Figure 1:**
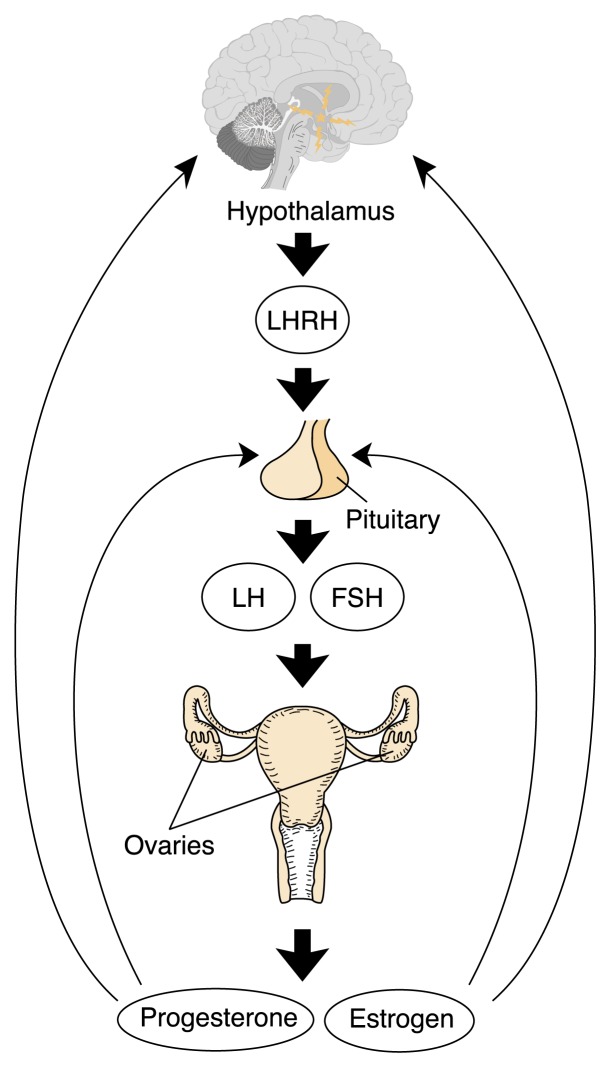
The female hypothalamic–pituitary–gonadal axis. The hypothalamus produces and secretes luteinizing hormone–releasing hormone (LHRH) into a system of blood vessels that link the hypothalamus and the pituitary gland. LHRH stimulates the pituitary gland by attaching to specific molecules (i.e., receptors). After the coupling of LHRH with these receptors, a cascade of biochemical events causes the pituitary gland to produce and secrete two hormones, luteinizing hormone (LH) and follicle-stimulating hormone (FSH). LH and FSH are two of a class of hormones commonly known as gonadotropins. They are secreted into the general circulation and attach to receptors on the ovary, where they trigger ovulation and stimulate ovarian production of the hormones estrogen and progesterone. These female hormones cause monthly menstrual cycling and have multiple effects throughout the body. In particular, estrogen has profound effects on the skeletal system and is crucial to maintaining normal bone health (Kanis 1994).

The female reproductive system includes three basic components: a brain region called the hypothalamus; the pituitary gland, located at the base of the brain; and the ovaries (Molitch 1995). These three components together make up the female hypothalamic–pituitary–gonadal (HPG) axis. This system is described in figure 1.

### Normal Mammalian Puberty

**Figure 2:**
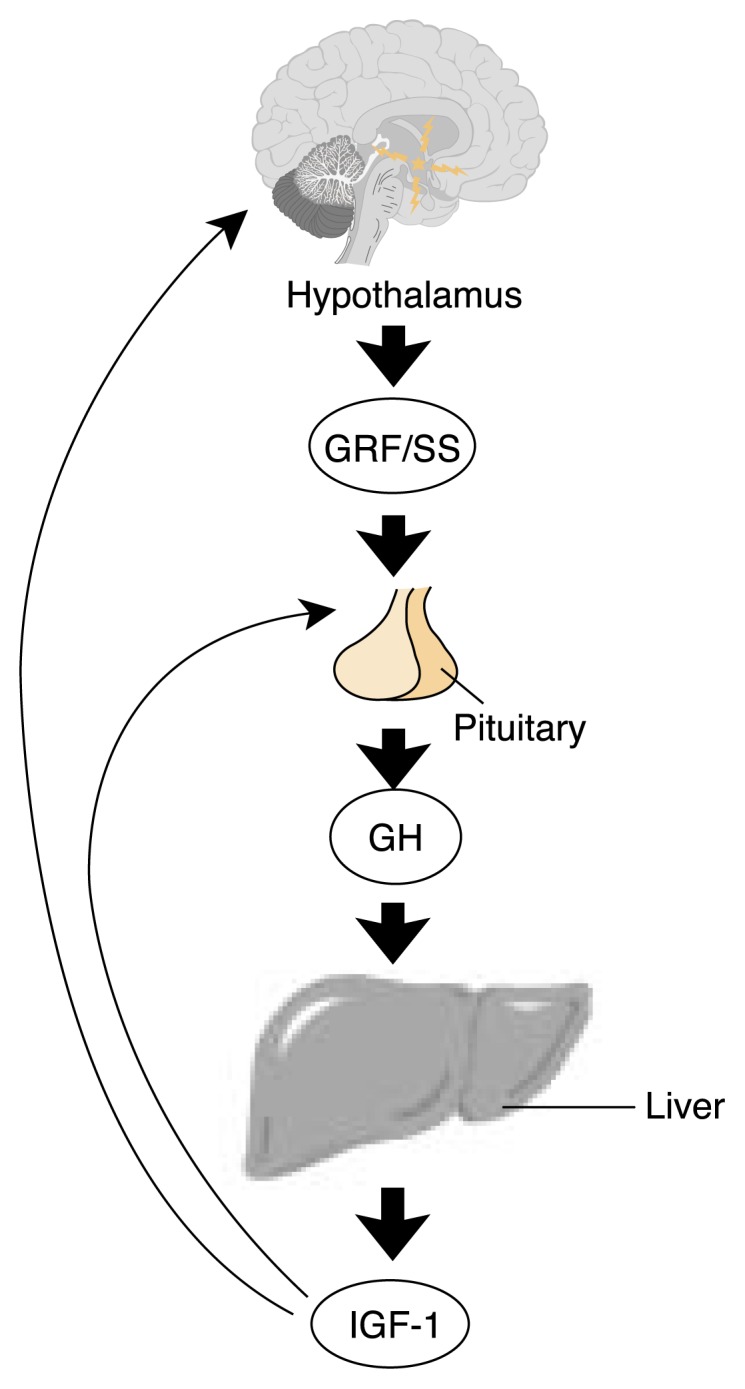
The female growth hormone–insulin-like growth factor (GH–IGF) axis. During puberty, there is a marked increase in growth hormone (GH) secretion from the pituitary as well as an increase in the secretion of the gonadotropins (Mauras et al. 1996). Like the HPG axis, GH secretion is regulated by interaction between the hypothalamus, pituitary, and a variety of organs, mainly the liver (Molitch 1995). The hypothalamus produces and secretes growth hormone–releasing factor (GRF) and the hormone somatostatin (SS) into the blood vessels linking the hypothalamus and pituitary. GRF stimulates GH synthesis and secretion, and SS inhibits GH secretion. GH, secreted into the general circulation, in turn stimulates the synthesis and secretion of the growth-stimulating hormone insulin-like growth factor 1 (IGF–1) in the liver and other organs. IGF–1 mediates many of the growth effects of GH. It also acts as an operative in a negative feedback loop, diminishing GH secretion by actions at the hypothalamus and pituitary. At the hypothalamus, IGF–1 stimulates SS and inhibits GRF release, and at the pituitary, IGF inhibits GH response to GRF. However, despite this negative feedback relationship, the only physiologic situation where both GH and IGF–1 are elevated is normal puberty.

Puberty is the dramatic awakening of the HPG axis, resulting in marked alterations in hormonal activity (especially the pituitary and gonadal hormones), physiologic processes (such as reproduction and growth), and behavior. It is generally accepted that this results from the activation of the hypothalamic secretion of luteinizing hormone–releasing hormone (LHRH), which in turn stimulates the pituitary secretion of luteinizing hormone (LH) and follicle-stimulating hormone (FSH), which leads to maturation and function of the ovaries (Mauras et al. 1996; Veldhuis 1996; Apter 1997). Because, like most hormones, LHRH is secreted episodically in pulses, rather than continuously, puberty has been viewed as an awakening of the LHRH pulse generator. Puberty is marked not only by the activation of reproductive processes but also by a growth spurt. The accompanying hormonal changes are depicted in figure 2.

The increased HPG activity and increased growth hormone (GH) secretion that occur during puberty are functionally interrelated, in that a variety of human and animal data have shown that the form of estrogen known as estradiol markedly stimulates the secretion of GH (Mauras et al. 1996). Moreover, the growth-stimulating hormone insulin-like growth factor 1 (IGF–1) can stimulate LHRH (Hiney et al. 1998). Thus, the HPG axis is activated, leading to both sexual maturation and a growth spurt, via estrogen’s stimulatory effects on the GH–IGF axis.

Pubertal development is influenced not only by HPG and GH–IGF activities but also by the opioid pathway. Endogenous opioid peptides (EOPs) are natural chemicals found in the body that act like opiates. There are three major EOPs, products of three separate genes. The major peptide in the female reproductive system is beta-endorphin, which is made in the hypothalamus as well as throughout the brain and in the pituitary. Hypothalamic beta-endorphin restrains the secretion of hypothalamic LHRH and inhibits the HPG axis. Compounds such as naloxone and naltrexone that block the effect of beta-endorphin are known as opiate antagonists. These compounds have been widely used to study the mechanisms of opioid inhibition of the HPG axis. In early puberty, naloxone administration does not change LH levels, indicating that normally during this time, little opioid inhibition of the HPG axis occurs (Petraglia et al. 1986; Genazzani et al. 1997). However, the situation changes in late puberty, when naloxone does normally evoke an LH response, indicating that opioid inhibition of the HPG axis increases during puberty. However, low opioid inhibition of the HPG axis in early puberty allows for or permits the activation of the HPG axis, which is the neuroendocrine hallmark of puberty. A variety of data indicate that opioid inhibition of LHRH release depends on the presence of gonadal steroids, so that the activation of the HPG axis during puberty leads to increased gonadal steroid levels, resulting in increased opioid inhibition of LHRH release in a classic negative feedback loop (Bhanot and Wilkinson 1983; Genazzani et al. 1990).

### Normal Female Cycle: Human and Rat

**Figure 3:**
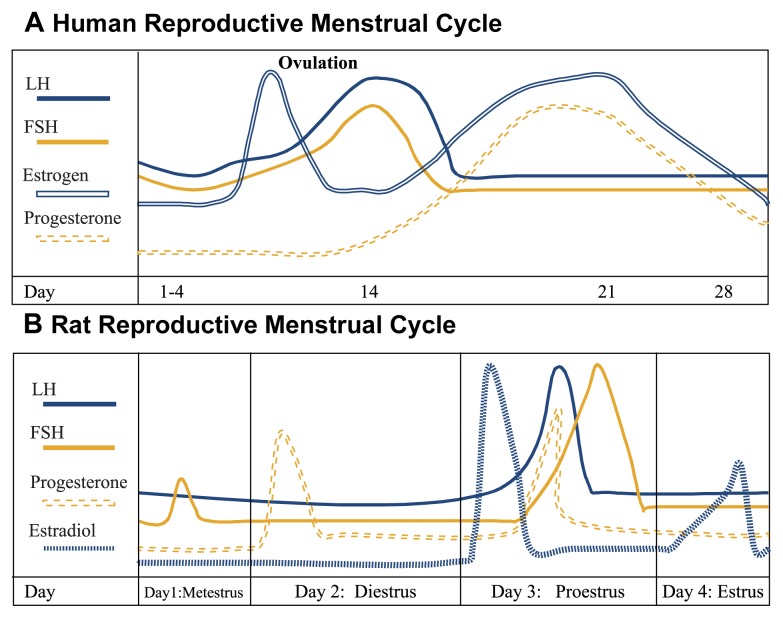
(A) The human reproductive cycle. A typical human reproductive menstrual cycle lasts 28 days, with ovulation occurring at midpoint, at day 14. The first day of vaginal bleeding is day 1. The first phase of the cycle is the follicular phase, during which estrogen and progesterone levels are very low. At approximately day 12, estrogen levels surge, causing increased secretion of pituitary LH and FSH, with levels peaking on day 14. This LH/FSH surge results in ovulation, sustained elevation of ovarian estrogen, and a new increase in progesterone levels. During the postovulation period, called the luteal phase, estrogen and progesterone levels first rise, then fall back to very low levels, at which point the next menses starts. (B) The rat reproductive cycle. The rat cycle is much shorter than the human cycle, consisting of 4 to 5 days. Progesterone increases sharply, beginning early in the postovulation phase (i.e., diestrus*) on day 2 and drops sharply in late diestrus on day 2. At approximately noon of the start of the follicular phase (i.e., proestrus**), estrogen levels markedly surge, causing a rapid peaking of LH and FSH between about 4 p.m. to 6 p.m. of proestrus and an increased progesterone secretion. As in humans, the gonadotropin surge triggers ovulation. All these hormones return to baseline levels when ovulation occurs (i.e., estrus) on day 4. Finally there is a brief temporary peak of estradiol on the evening of estrus. * Diestrus is the luteal phase. ** Proestrus is the beginning of the follicular phase.

The typical human reproductive menstrual cycle encompasses a 28-day time-frame, with the first day of vaginal bleeding being day 1, and with ovulation occurring at midpoint, on day 14 (see figure 3A). The first phase of the cycle is the follicular phase, during which estrogen and progesterone levels are very low. During this time, the pituitary gonadotropins, primarily FSH, stimulate the maturation of ovarian follicles (i.e., the egg [ovum] and its surrounding estrogen- and progesterone-secreting cells). At approximately day 12, estrogen levels surge (known as the proestrous surge), signaling rapid follicular maturation and causing increased secretion of pituitary LH and FSH, with levels peaking on day 14. Estrogen does this (signaling and causing increased secretion) by sensitizing the pituitary gonadotropin-producing cells to the stimulatory effects of LHRH. This LH/FSH surge results in ovulation, sustained elevation of ovarian estrogen, and a new increase in progesterone levels. During the postovulation period, called the luteal phase, estrogen and progesterone levels first rise, then fall back to very low levels, at which point the next cycle starts. Estrogen and progesterone prepare the uterine wall for embryo implantation and growth, should pregnancy occur. Although the length of the follicular phase varies greatly between females, the length of the luteal phase is usually constant.

In contrast with the human cycle, the rat cycle is much shorter, consisting of 4 to 5 days (see figure 3B). Progesterone increases sharply beginning early in the postovulation phase (i.e., diestrus) on day 2 and drops sharply in late diestrus on day 2. At approximately noon of the start of the follicular phase (i.e., proestrus), estrogen levels markedly surge, causing a rapid peaking of LH and FSH between about 4 p.m. and 6 p.m. of proestrus and an increased progesterone secretion. As in humans, the gonadotropin surge triggers ovulation. All these hormones return to baseline levels when ovulation occurs (i.e., estrus) on day 4. Finally there is a brief temporary peak of estradiol the evening of estrus.

### Hormones in the Postmenopausal Female

**Figure 4:**
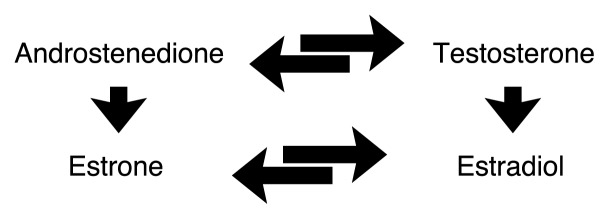
Synthesis of postmenopausal estrogens. Postmenopausal estrogens are synthesized from androgens (i.e., testosterone and androstenedione). In females, androgens are produced in the ovaries and the adrenal glands. They are transported in the bloodstream to body fat, where androstenedione is converted to estrone. Estrone replaces estradiol as the primary estrogen after menopause.

Estrogen production continues after the cessation of reproductive function, although estrogen levels are much lower. Postmenopausal estrogens are synthesized from androgens (i.e., testosterone and androstenedione) (see figure 4). In males, androgens are produced by the testes and are the primary reproductive hormones. In females, androgens are produced in the ovaries and the adrenal glands. They are transported in the bloodstream to body fat, where androstenedione is converted to estrone (Korenman et al. 1978). Estrone replaces estradiol as the primary estrogen after menopause. Estradiol levels are markedly lower in the menopausal female and are derived largely from the metabolism of estrone. Levels of testosterone and ovarian androstenedione also decrease after menopause, while adrenal androstenedione remains unchanged. The lack of ovarian hormones leads to a marked increase of FSH and LH.

## Alcohol’s Effects on Female Reproduction

The following section details alcohol’s effects on puberty, the female reproductive system, and postmenopause, as revealed by human and animal studies.

### Alcohol and Puberty

Rapid hormonal changes occurring during puberty make females especially vulnerable to the deleterious effects of alcohol exposure during this time. Thus, the high incidence of alcohol consumption among middle school and high school students in the United States is a matter of great concern. A national survey of students revealed that 22.4 percent of 8th graders and 50 percent of 12th graders reported consuming alcohol in the 30 days before the survey (Johnston et al. 2001).

Little research on the physiological effects of alcohol consumption during puberty has focused on human females. However, one study found that estrogen levels were depressed among adolescent girls ages 12 to 18 for as long as 2 weeks after drinking moderately (Block et al. 1993). This finding suggests the possibility that alcohol alters the reproductive awakening and maturation that marks puberty. Also, estrogen’s role in bone maturation raises the question of whether alcohol use during adolescence has long-term effects on bone health. Alcohol consumption during adolescence is known to affect growth and body composition, perhaps by altering food intake patterns while alcohol is being consumed (Block et al. 1991).

Most of the studies in this area have been done with animals, and this research has established that alcohol disrupts mammalian female puberty. Two decades ago, Van Thiel and colleagues (1978) showed that prepubertal rats fed alcohol as 36 percent of their calories for 7 weeks showed marked ovarian failure (based on structural and functional evaluation) compared with animals that did not receive alcohol but were fed the same number of total calories (i.e., pair-fed control subjects).

Subsequently, Bo and colleagues (1982) reported that vaginal opening, a well-characterized marker of puberty in the female rat, was delayed by alcohol administration. In a series of papers, Dees and colleagues (Dees et al. 1990, Dees and Skelley 1990) defined the hormonal changes responsible for this effect. Notably, alcohol caused an increase in hypothalamic levels of LHRH and a decrease in levels of LH in the bloodstream (Rettori et al. 1987; Dees et al. 1990). Taken together, these findings suggested that an alcohol-induced decrease in hypothalamic LHRH secretion (leading to the increased hypothalamic content) accounts for the decrease in LH. Indeed, Hiney and Dees (1991) demonstrated that alcohol was able to reduce LHRH secretion from hypothalamic slices taken from prepubertal female rats. In addition to the LHRH/LH findings, the authors reported an alcohol-induced increase in hypothalamic levels of growth hormone–releasing factor (GRF) coupled with a decrease in bloodstream levels of GH (Dees and Skelley 1990). Analogous to the interpretation of the LHRH/LH data above, these data suggested that alcohol led to a decreased GH secretion by decreasing GRF release from the hypothalamus. Levels of the hormone somatostatin (SS) were not affected by alcohol administration.

GH mediates many of its growth effects via stimulation of the synthesis and secretion of IGF–1. As would be anticipated from the fact that alcohol decreases GH, alcohol also decreases IGF–1 (Srivastava et al. 1995; Steiner et al. 1997), which could account, in part at least, for impaired growth in animals given alcohol, despite pair-feeding procedures.

A recent study in developing Rhesus monkeys has demonstrated detrimental effects of alcohol on the activation of hormone secretion that accompanies female puberty (Dees et al. 2000). Although alcohol did not affect the age of menarche in this mammalian model, the interval between subsequent menstruations was lengthened, showing that alcohol affected the development of a regular monthly pattern of menstruation. The authors suggest that the growth spurt and normal timing or progression of puberty may be at risk in human adolescents consuming even relatively moderate amounts of alcohol on a regular basis.

Research with adult rats has shown that alcohol increases opioid activity in the brain (Froehlich 1993). If this is true in the pubertal animal as well, it may represent one of the mechanisms by which alcohol disrupts puberty. As stated above, puberty is markedly delayed in prepubertal female rats given alcohol, as manifested by delayed vaginal opening. However, when these rats are given naltrexone to block opioid receptors, the alcohol-induced delay in vaginal opening is completely prevented (Emanuele et al. 2002). This suggests that at least part of the alcohol-induced pubertal delay is attributable to increased opioid restraint of the normal progression of development.

Investigators have not addressed the implications of alcohol exposure during puberty for subsequent fertility. Future research may examine, for example, whether alcohol exposure during puberty alters chromosomes, leading to deformities in offspring.

### Alcohol and the Female Reproductive System

Alcohol markedly disrupts normal menstrual cycling in female humans and rats. Alcoholic women are known to have a variety of menstrual and reproductive disorders, from irregular menstrual cycles to complete cessation of menses, absence of ovulation (i.e., anovulation), and infertility (reviewed in Mello et al. 1993). Alcohol abuse has also been associated with early menopause (Mello et al. 1993). However, alcoholics often have other health problems such as liver disease and malnutrition, so reproductive deficits may not be directly related to alcohol use.

In human females, alcohol ingestion, *even in amounts insufficient to cause major damage to the liver or other organs*, may lead to menstrual irregularities (Ryback 1977). It is important to stress that alcohol ingestion at the wrong time, even in amounts insufficient to cause permanent tissue damage, can disrupt the delicate balance critical to maintaining human female reproductive hormonal cycles and result in infertility. A study of healthy nonalcoholic women found that a substantial portion who drank small amounts of alcohol (i.e., social drinkers) stopped cycling normally and became at least temporarily infertile. This anovulation was associated with a reduced or absent pituitary LH secretion. All the affected women had reported normal menstrual cycles before the study (Mendelson and Mello 1988). This finding is consistent with epidemiologic data from a representative national sample of 917 women, which showed increased rates of menstrual disturbances and infertility associated with increasing self-reported alcohol consumption (Wilsnack et al. 1984). Thus, alcohol-induced disruption of female fertility is a clinical problem that merits further study.

Several studies in both rats and monkeys have demonstrated alcohol-induced reproductive disruptions similar to those seen in humans. These studies have provided some information on how both acute and chronic alcohol exposure can alter the animals’ reproductive systems. For example, acute alcohol exposure in female rats has been found to disrupt female cycling (LaPaglia et al. 1997). Acute alcohol exposure given as a bolus (i.e., an injection of a high dose) to mimic binge drinking has been reported to disrupt the normal cycle at the time of exposure, with a return to normal by the following cycle (Alfonso et al. 1993). A study of female rats fed alcohol or a control diet for 17 weeks starting at young adulthood (comparable in age to a 21-year-old woman) found that alcohol did not lead to anovulation but rather to irregular ovulation (Krueger et al. 1983; Emanuele et al. 2001). Other investigators (Gavaler et al. 1980), however, have reported that the ovaries of alcohol-exposed female rats were infantile, showing no evidence of ovulation at all, and uteri appeared completely estrogen deprived. The different outcomes described in these studies may be attributable to the different strains of rats used. It should be noted, however, that if enough alcohol is given, cyclicity can be completely abolished, as demonstrated in dose-response studies (i.e., studies that examined the varying responses to increasing doses of alcohol) (Cranston 1958; Eskay et al. 1981; Rettori et al. 1987).

Recently investigators have provided several insights into the possible mechanisms underlying alcohol’s disruption of the female cycle in the rat model. First, research shows that alcohol-fed rats have a temporary elevation of estradiol (Emanuele et al. 2001). Human studies have produced similar findings (Mello et al. 1993). The effects of estrogen on reproductive cyclicity are complex. In some situations, estrogen stimulates the hypothalamic–pituitary unit (Tang et al. 1982); in other situations, it is inhibitory. This short-term elevation in estradiol may be part of the mechanism underlying the alcohol-induced alterations in estrous cycling.

Second, alcohol consumption temporarily increases testosterone levels (Sarkola et al. 2001). Because testosterone is a well-known suppressor of the hypothalamic–pituitary unit, an increase in testosterone could therefore disturb normal female cycling.

Third, both acute and chronic alcohol treatments have been shown to decrease levels of IGF–1 in the bloodstream. This is potentially relevant, because IGF–1, in addition to its well-known effects in promoting some of the growth effects of GH, has reproductive effects as well (Mauras et al. 1996). Specifically, IGF–1 has been shown to evoke LHRH release in female rats, as demonstrated by Hiney and colleagues (1991, 1996) both in animal studies and in tissue culture studies. Moreover, in acute alcohol studies, the ability of IGF–1 to increase LH was blocked by alcohol (Hiney et al. 1998). Thus, alcohol may disrupt reproductive cyclicity by diminishing IGF–1 neuroendocrine stimulation.

### Alcohol in the Postmenopausal Female

Purohit (1998) and Longnecker and Tseng (1998), in recent reviews of the research on alcohol’s effects on post-menopausal females, found some evidence that acute alcohol exposure results in a temporary increase in estradiol levels in menopausal women on hormone replacement therapy (HRT). This increase may be attributed to impaired estradiol metabolism, with decreased conversion of estradiol to estrone (Purohit 2000). Interestingly, alcohol exposure had no effect on estradiol levels in women who were not receiving HRT, or on estrone levels in either group of women (Purohit 1998; Longnecker and Tseng 1998). No controlled studies have examined the effect of chronic alcohol consumption among postmenopausal women, but research using self-report data has shown that alcohol use in postmenopausal women has mixed effects on estradiol levels in women not on HRT. In contrast, women receiving HRT had lower levels of estradiol when their alcohol consumption was high (Johannes et al. 1997). Thus, the amount of alcohol consumed appears to be an important variable in studies of hormone levels in postmenopausal women who consume alcohol. Other studies have demonstrated that alcohol consumption after menopause is unrelated to levels of testosterone and androstenedione (Gavaler et al. 1993).

These epidemiological studies do not address confounding factors such as malnutrition, medications, and other medical problems. Also, drinking patterns, type of alcohol consumed, and time elapsed since last drinking episode prior to testing are not standardized. Overall, the data suggest that alcohol does not affect estrone levels but may increase estradiol. Further studies in this area are clearly needed.

The literature provides little information on the effects of alcohol in the older female rat model. One study of rats whose ovaries had been surgically removed, mimicking the human menopausal state, demonstrated that heavy chronic alcohol exposure (4.4 grams of alcohol/kg body weight/day for 10 weeks) was able to increase estrogen levels (Gavaler and Rosenblum 1987). In female rats, the available data are not adequate to determine the impact of alcohol on the conversion of androgens to estrogens (i.e., aromatization). Further studies are necessary to investigate the effects of moderate versus heavy doses of alcohol on this process (Purohit 2000).

As reviewed above, alcohol use has been shown to affect female puberty, reproductive function, and hormonal levels in postmenopausal women. Through its effects on these stages of life, alcohol use can influence bone health, as described next.

### Effects of Alcohol-Induced Reproductive Dysfunction on the Skeleton

Heavy alcohol use is a recognized risk factor for osteoporosis in humans (Singer 1995). Human observational studies have not clearly indicated whether the osteoporosis seen in people who used alcohol was caused by alcohol itself or by attendant nutritional deficiencies. Well-controlled experiments, however, have demonstrated that alcohol itself can cause osteoporosis in growing and adult animals (Sampson et al. 1996, 1997; Hogan et al. 1997, 2001; Wezeman et al. 1999).

Osteoporosis has many negative consequences. It increases vulnerability to fractures, which can lead to immobilization and subsequent depression, markedly decreased quality of life, loss of productive work time, bed sores, sepsis, and more osteoporosis. Risk for osteoporosis is in part related to low peak bone mass (Singer 1995): the lower the peak bone mass, the greater the risk for osteoporosis. Active bone growth occurs during puberty, and alcohol’s disruption of bone development in animals (Sampson et al. 1996, 1997; Hogan et al. 1997; Wezeman et al. 1999) may cause lifelong osteoporosis in animals exposed to alcohol at a young age (Sampson et al. 1998).

Two important processes are necessary to maintain normal bone integrity: the destruction of old bone, known as resorption, and the production of new bone, known as formation. Estrogen helps to regulate bone turnover and plays a significant part in the maintenance of skeletal mass, perhaps through modulating local factors involved in bone growth and maintenance, including messenger molecules known as cytokines and growth factors (Kimble 1997). The interplay of numerous local and systemic factors (such as estrogens and androgens) ultimately determines the net effect of these substances on skeletal tissue. Whereas in the normal adult a balance of these many factors maintains skeletal mass (Frost 1986), a positive balance (formation relative to resorption) characterizes bone growth. In pathological conditions (e.g., chronic heavy alcohol consumption), the normal relationship between bone formation and resorption is altered, leading to osteoporosis. Alcohol abuse contributes to bone weakness, increasing the risk of fracture (Orwoll and Klein 1995).

Alcoholics have reduced bone mass, which is evident in the loss of bone tissue in the spine and iliac crest. In experimental animals, the reduced bone mass is also evident in the lower extremities. There is general agreement that alcohol consumption decreases bone formation through a decrease in the number of bone cells responsible for bone formation (i.e., osteoblasts) (Klein 1997), which is accompanied by a reduction in bone cell function (Klein 1997).

In some of the studies reviewed above, heavy alcohol consumption has been found to increase estrogen production, which should protect bone from the development of osteoporosis. Yet, despite this increase in estrogen, alcohol consumption leads to accelerated bone loss. Alcohol does not accelerate the bone loss associated with gonadal insufficiency and may reduce the number of bone-resorbing cells (i.e., osteoclasts) (Kidder and Turner 1998). Resolving this apparent paradox should be an interesting focus of future research.

Gender-specific skeletal changes in relation to alcohol use during reproductive maturation have not been sufficiently addressed in research. The functional capacity of bone cells in estrogen or androgen environments differs, and bone mass as a correlate of muscle mass differs between genders. It is reasonable to conclude that the response of bone to alcohol consumption will differ for males and females, particularly when the hormonal environment is established at puberty. It is important to investigate whether or not, in humans, alcohol-induced osteoporosis beginning in puberty is lifelong.

## Summary

As reviewed here, research shows that alcohol use negatively affects puberty in females, disrupts normal menstrual cycling and reproductive function, and alters hormonal levels in postmenopausal women. These effects of alcohol use can also have important consequences for bone health. Further research is needed to determine the mechanisms of these effects and to design strategies to prevent them.
